# Crystal structure and biochemical characterization of the recombinant ThBgl, a GH1 β-glucosidase overexpressed in *Trichoderma harzianum* under biomass degradation conditions

**DOI:** 10.1186/s13068-016-0487-0

**Published:** 2016-03-22

**Authors:** Clelton A. Santos, Letícia M. Zanphorlin, Aline Crucello, Celisa C. C. Tonoli, Roberto Ruller, Maria A. C. Horta, Mario T. Murakami, Anete Pereira de Souza

**Affiliations:** Centro de Biologia Molecular e Engenharia Genética, Universidade Estadual de Campinas, Campinas, SP Brazil; Laboratório Nacional de Ciência e Tecnologia do Bioetanol, Centro Nacional de Pesquisa em Energia e Materiais, Campinas, SP Brazil; Laboratório Nacional de Biociências, Centro Nacional de Pesquisa em Energia e Materiais, Campinas, SP Brazil; Departamento de Biologia Vegetal, Instituto de Biologia, Universidade Estadual de Campinas, Campinas, SP Brazil

**Keywords:** *Trichoderma harzianum*, β-Glucosidase, Overexpression, Enzyme prospection, Biomass degradation

## Abstract

**Background:**

The conversion of biomass-derived sugars via enzymatic hydrolysis for biofuel production is a challenge. Therefore, the search for microorganisms and key enzymes that increase the efficiency of the saccharification of cellulosic substrates remains an important and high-priority area of study. *Trichoderma harzianum* is an important fungus known for producing high levels of cellulolytic enzymes that can be used for cellulosic ethanol production. In this context, β-glucosidases, which act synergistically with cellobiohydrolases and endo-β-1,4-glucanases in the saccharification process, are potential biocatalysts for the conversion of plant biomass to free glucose residues.

**Results:**

In the present study, we used RNA-Seq and genomic data to identify the major β-glucosidase expressed by *T. harzianum* under biomass degradation conditions. We mapped and quantified the expression of all of the β-glucosidases from glycoside hydrolase families 1 and 3, and we identified the enzyme with the highest expression under these conditions. The target gene was cloned and heterologously expressed in *Escherichia coli*, and the recombinant protein (rThBgl) was purified with high yields. rThBgl was characterized using a comprehensive set of biochemical, spectroscopic, and hydrodynamic techniques. Finally, we determined the crystallographic structure of the recombinant protein at a resolution of 2.6 Å.

**Conclusions:**

Using a rational approach, we investigated the biochemical characteristics and determined the three-dimensional protein structure of a β-glucosidase that is highly expressed by *T. harzianum* under biomass degradation conditions. The methodology described in this manuscript will be useful for the bio-prospection of key enzymes, including cellulases and other accessory enzymes, for the development and/or improvement of enzymatic cocktails designed to produce ethanol from plant biomass.

**Electronic supplementary material:**

The online version of this article (doi:10.1186/s13068-016-0487-0) contains supplementary material, which is available to authorized users.

## Background

β-Glucosidases (EC 3.2.1.21) catalyze the conversion of cellobiose to glucose monomers, which can then be fermented to produce ethanol [1–3]. Together with cellobiohydrolases (EC 3.2.1.91) and endo-β-glucanases (EC 3.2.1.4), β-glucosidases form a powerful cellulolytic system present in all microorganisms that use cellulose as a substrate [1]. The cellobiohydrolases, depending on their specificity, can generate cellobiose by attacking either the reducing or non-reducing ends of cellulose chains, while endo-β-glucanases hydrolyze the internal β-1,4-glucosyl linkages. The β-glucosidases are key enzymes that act at the final stage of plant biomass hydrolysis and are potential candidates for biotechnological applications [1–3].

β-glucosidases play an important role in the saccharification of cellulosic substrates because these enzymes decrease the inhibitory effect of cellobiose on the enzymatic activity of the cellobiohydrolases and endo-β-glucanases [4–6]. However, many known β-glucosidases are sensitive to the glucose product or inhibited by their cello-oligosaccharide substrates [3, 6, 7]. Thus, the enzymatic degradation of cellulosic biomass is a synergistic process, and each enzyme catalyzes an important step in the continuous and complete breakdown of cellulose [5, 8, 9]. Therefore, a better understanding of the complete hydrolytic process can be achieved by studies that dissect the structure and function of the enzymes in this pathway.

The Carbohydrate-Active enZymes (CAZy) database (http://www.cazy.org) mainly groups the β-glucosidases into glycosyl hydrolase (GH) families 1 and 3 [10]. This grouping is based on structural characteristics, especially those related to the mechanism of enzymatic catalysis. Although, both act on similar substrates, the GH1 β-glucosidases use a Glu residue as the catalytic nucleophile, whereas the GH3 β-glucosidases use an Asp residue as the nucleophile [11, 12]. The protein data bank (PDB; http://www.rcsb.org/pdb/home/home.do) contains many β-glucosidase structures derived from prokaryotic and eukaryotic organisms. The elucidation of the structure of β-glucosidase enzymes has led to a better understanding of how to improve the enzymatic degradation of biomass and constitutes an important field of study.

To increase the efficiency of the saccharification of cellulosic substrates, enzymes with promising features for industrial applications are continually sought [13–16]. In particular, enzymes that are heavily used by microorganisms under specific conditions are promising targets for practical applications. With advances in RNA sequencing (RNA-Seq) technology [17], the transcriptional profiles of *Trichoderma harzianum*, an important fungus with an efficient cellulase machinery, and other cellulolytic microorganisms are currently available [18–22]. This technology can facilitate the discovery of the differentially expressed genes under specific metabolic conditions.

In this study, by screening RNA-Seq libraries, we identified a GH1 β-glucosidase that was highly expressed by the *T. harzianum* strain IOC-3844 under biomass degradation conditions. The target gene was cloned and heterologously expressed in *Escherichia coli*. The crystallographic structure of the purified recombinant protein was determined at a resolution of 2.6 Å. Spectroscopic, hydrodynamic, and biochemical studies were conducted with the recombinant enzyme. Our work sheds new light on the strategies for enzyme bio-prospection and on the potential use of key proteins involved in the enzymatic hydrolysis of cellulose.

## Results and discussion

### Mapping and quantification of the expression of β-glucosidases from *T. harzianum* using RNA-Seq data

We used RNA-Seq libraries to access the reads per kilo base per million of mapped reads (RPKM) for the multiple β-glucosidases expressed by *T. harzianum* under biomass degradation conditions. Once the transcriptional profile data from an organism under a certain condition is known, the levels of any gene can be determined (mapping and quantifying) within the limitations/conditions of the original experiments [17, 23]. The transcriptome profile of *T. harzianum* IOC-3844 cultured in the presence of lactose (LAC), crystalline cellulose (CEL), or delignified sugarcane bagasse (DSB) was reported by Horta et al. [18]. These data enable the identification of the set of genes involved in biomass degradation and thus provide a powerful tool for future studies.

**Fig. 1:**
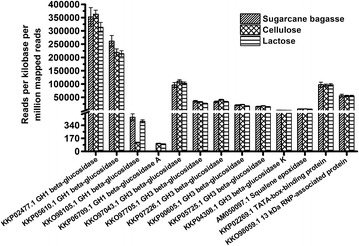
Gene mapping and quantification of the expression of β-glucosidases from *T. harzianum* using RNA-Seq data. The genes encoding the GH1 and GH3 β-glucosidases that were used for read mapping were identified from the assembled genome of the *T. harzianum* T6776 strain (GenBank access number JOKZ00000000.1) (Additional file 1: Table S1) and compared to the RNA-Seq libraries from *T. harzianum* IOC-3844 cultured with LAC, CEL, or DSB. The squalene epoxidase (GenBank: AM050097.1), TATA box-binding protein (GenBank: KKP02269.1) and the 13-kDa ribonucleoprotein (RNP)-associated protein (GenBank: KKO98059.1) sequences were used for the normalization of gene expression. *RPKM* reads per kilobase per million mapped reads

In this study, we initially identified all of the GH1 and GH3 β-glucosidases using the assembled genome of the *T. harzianum* T6776 strain (GenBank access number JOKZ00000000.1) [24]. We then used these sequences to map and quantify the expression of these genes using *T. harzianum* IOC-3844 transcriptome data. Four GH1 and six GH3 β-glucosidase sequences were found in the *T. harzianum* T6776 genome (Additional file 1: Table S1) and used in the mapping experiments. The mapping results revealed that *T. harzianum* IOC-3844 had higher levels of the GH1 β-glucosidases than the GH3 β-glucosidases (Fig. 1). Among the four GH1 β-glucosidases, two enzymes (KKP02477.1 and KKP05610.1) had the highest overall expression, while the other two enzymes (KKO98105.1 and KKP06709.1) exhibited basal levels of expression. Analyses of the amino acid sequences of these proteins showed sequence identities ranging from 27.46 to 53.78 % (Additional file 2: Table S2). Notably, the sequence for KKP02477.1, the enzyme with the highest expression among all the β-glucosidases studied (approximately 400,000 RPKM), was the target of our study. However, this protein in *T. harzianum* IOC-3844 (GenBank: KU201604.1) contains a change of a glutamine residue by a glutamic acid at position 335, and a methionine is replaced by an isoleucine residue at position 462, sharing 99.57 % identity.

Although six GH3 β-glucosidases were found in the genome of *T. harzianum*, these enzymes are expressed at similar levels regardless of treatment conditions (LAC, CEL, or DSB) (Fig. 1). The variation in the GH3 β-glucosidase amino acid sequences was investigated, and the sequence identities ranged from 23.93 to 50.00 % (Additional file 3: Table S3). The squalene epoxidase (GenBank: AM050097.1), TATA box-binding protein (GenBank: code: KKP02269.1), and 13-kDa ribonucleoprotein (RNP)-associated protein (GenBank: KKO98059.1) sequences were used as housekeeping genes for the mapping experiments (Fig. 1).

In general, approaches utilizing RNA-Seq libraries may enable the discovery of promising enzymes for industrial applications. Thus, this may be a useful tool to search for highly expressed targets and other accessory enzymes that can improve cellulose hydrolysis during biomass degradation.

### Spectroscopic and hydrodynamic characterization of purified rThBgl

After the β-glucosidase with the highest level of expression in *T. harzianum* under biomass degradation conditions was identified, gene cloning and heterologous protein expression and purification were carried out. rThBgl (468 amino acid residues, 53.2 kDa and theoretical isoelectric point of 5.1) was successfully expressed using *E. coli* as a host. The recombinant protein was purified using two chromatographic steps: nickel affinity (using the N-terminal His_6_-tag added by the pET28a expression vector) and size-exclusion chromatography (SEC). Approximately 35.5 mg of protein with a purity greater than 95 % based on 12 % SDS-PAGE was obtained per liter of bacterial culture.

Studies on the protein expression and purification of β-glucosidases from different *Trichoderma* fungus strains have been performed [25–34]; however, most of these studies used eukaryotic expression systems, in particular *Trichoderma reesei* and *Pichia pastoris*. In the current study, we overexpressed and purified rThBgl using an *E. coli* host. This method produces high yields of the recombinant protein, which suggests that it can potentially be used in industrial processes for cellulosic ethanol production [35–38].

**Fig. 2:**
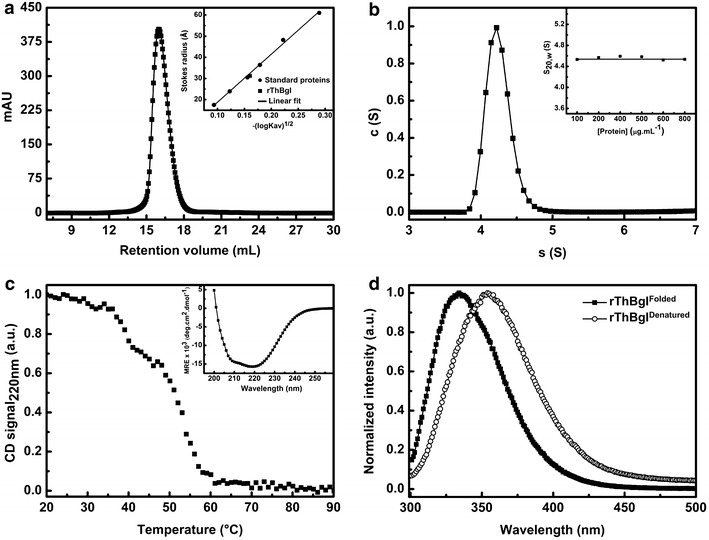
Hydrodynamic and spectroscopic features of rThBgl. **a** Analytical SEC experiments with purified rThBgl. The protein elution profiles were used to estimate the *R*_s_. The detailed procedure is described in the “Methods” section. *Inset* Estimation of the rThBgl Stokes radii as a function of the values of −(logK_av_)^1/2^ using standard proteins. **b** Sedimentation velocity AUC experiments of rThBgl using a concentration range of 100–800 μg mL^−1^ in buffer C. The figure presents the c(*S*) distribution of the experiment at 800 μg mL^−1^. Even at high concentrations, all sedimentation profiles exhibited only one species. *Inset*: Dependence of rThBgl s_20,w_ (*S*) as a function of protein concentration. We calculated an $S_{20,w}^{0}$ (*S*) of 4.53 ± 0.03 S. The results in A and B together indicate that rThBgl is a monomer in solution (Table 1). **c** rThBgl thermal-induced unfolding measurements followed by CD. The unfolding experiments were measured at 220 nm from 20 to 90 °C with a 1-mm-path length cell using 8 µM rThBgl in buffer A. rThBgl had a *Tm* of 49 ± 1 °C. The typical α-helix-rich CD spectrum of rThBgl is presented in the inset of panel **c**. **d** Fluorescence emission spectra excited at 280 nm of folded and denatured states of rThBgl. To ensure complete protein denaturation, rThBgl was incubated with a freshly prepared solution of GdnHCl at a final concentration of 6 M for 1 h before the fluorescence experiments. The intrinsic fluorescence emission spectra were collected from 300 to 500 nm and revealed a clear shift of 334–354 nm between the folded and denatured rThBgl samples, respectively

The hydrodynamic and spectroscopic properties of the purified rThBgl were assessed (Table 1, Fig. 2). For the hydrodynamic analysis, analytical SEC and analytical ultracentrifugation (AUC) techniques were employed. The results of the analytical SEC experiments revealed that rThBgl was eluted as a unique peak with a retention time that corresponded to an apparent molecular mass (MM_app_) of 54.72 ± 3 kDa (Fig. 2a). Based on the analytical SEC elution profile and standard proteins with known Stokes radii (see “Methods” section), the Stokes radius (*R*_s_) and frictional ratio (ƒ/ƒ_0_) of rThBgl were estimated to be 31.39 Å and 1.25 ± 0.2, respectively (Table 1, Fig. 2a). The results from the AUC experiments corroborated the analytical SEC data; during sedimentation, rThBgl appeared as a single species with an $S_{20,w}^{0}$ and experimental molecular mass (MM_exp_) of 4.53 ± 0.03 *S* and 58.26 ± 7 kDa, respectively. The ƒ/ƒ_0_ value was 1.23 ± 0.05 (Table 1, Fig. 2b). The analytical SEC and AUC data suggest that purified rThBgl behaves as a monodisperse monomer in solution. A similar finding was previously reported for other β-glucosidases from *Trichoderma* spp. [25, 30]. In addition, based on a ƒ/ƒ_0_ of 1.2, the protein is expected to have a globular shape [39].

**Table 1:** Hydrodynamic and spectroscopic properties of purified rThBgl

Technique	Property
Predicted hydrodynamic data^a^	MM_pred_ = 53.24 kDa
	*R*_0_ = 25.15 Å
Analytical SEC	MM_app_ = 54.72 ± 3 kDa
	*R*_s_ = 31.39 Å
	*ƒ*/*ƒ*_0_^b^ = 1.25 ± 0.2
AUC^c^	$S_{20,w}^{0}$ = 4.53 ± 0.03 S
	MM_exp_ = 58.26 ± 7 kDa
	*ƒ*/*ƒ*_0_ = 1.23 ± 0.05
CD^d^	*α*-Helix = 35 ± 4 %
	*β*-Sheet = 10 ± 2 %
CD thermal-induced unfolding	Tm = 49 ± 1 °C
Fluorescence	*λ*_max_^rThBgl−folded^ = 334 ± 1 nm
	〈λ〉^rThBgl−folded^ = 353 ± 1 nm
	*λ*_max_^rThBgl−denatured^ = 354 ± 2 nm
	〈λ〉^rThBgl−denatured^ = 363 ± 1 nm

*MM*_*prep*_ predicted molecular mass, *MM*_*exp*_ experimental molecular mass, *MM*_*app*_ apparent molecular mass

^a^Predicted data from the amino acid sequence of rThBgl using the Sednterp server (http://sednterp.unh.edu/)

^b^From the ratio of *R*_s_:*R*_0_

^c^Obtained from a SedFit analysis

^d^Secondary structure generated by deconvolution of the experimental CD spectra using the CDNN Deconvolution program

The secondary structure composition and tertiary folding of rThBgl were assessed by circular dichroism (CD) and fluorescence spectroscopy, respectively. rThBgl had a typical CD spectrum for an α/β-folded protein (Fig. 2c, inset) as previously described for the GH1 β-glucosidase family [40, 41]. The α-helix and β-sheet contents estimated from the deconvolution of the rThBgl spectrum were 35 ± 4 and 10 ± 2 %, respectively (Table 1), which are similar to those observed in the crystallographic structure. We also used the CD technique to evaluate the thermal-induced unfolding properties of rThBgl. These data revealed that rThBgl underwent conformational changes at approximately 35 °C and was completely unfolded at ~61 °C, resulting in a melting temperature (Tm) of 49 ± 1 °C (Table 1, Fig. 2c), which is consistent with the effect of temperature on the enzymatic activity. The results of thermal-induced unfolding were similar to those obtained by differential scanning calorimetry (data not shown) and confirmed the thermal stability properties of rThBgl.

Using the intrinsic fluorescence characteristics of tryptophan residues, the local tertiary structure of rThBgl was investigated in its folded and denatured states. A clear difference in the fluorescence emission spectra between the folded and denatured forms was observed (Fig. 2d). The maximum emission wavelength (*λ*_max_) and spectral center of mass 〈λ〉 for the folded rThBgl were 334 ± 1 and 353 ± 1 nm, respectively, while the denatured form of rThBgl exhibited a *λ*_max_ and 〈λ〉 of 354 ± 2 nm and 363 ± 1 nm, respectively (Table 1). rThBgl has 12 tryptophan residues distributed along the protein amino acid sequence; therefore, the changes observed with fluorescence spectroscopy indicate that the recombinant protein was produced in a folded state. The use of a denaturing agent, such as GdnHCl, exposed some of these tryptophan residues to the solvent, thus disrupting the local tertiary structure.

### The kinetic and biochemical properties of rThBgl

**Fig. 3:**
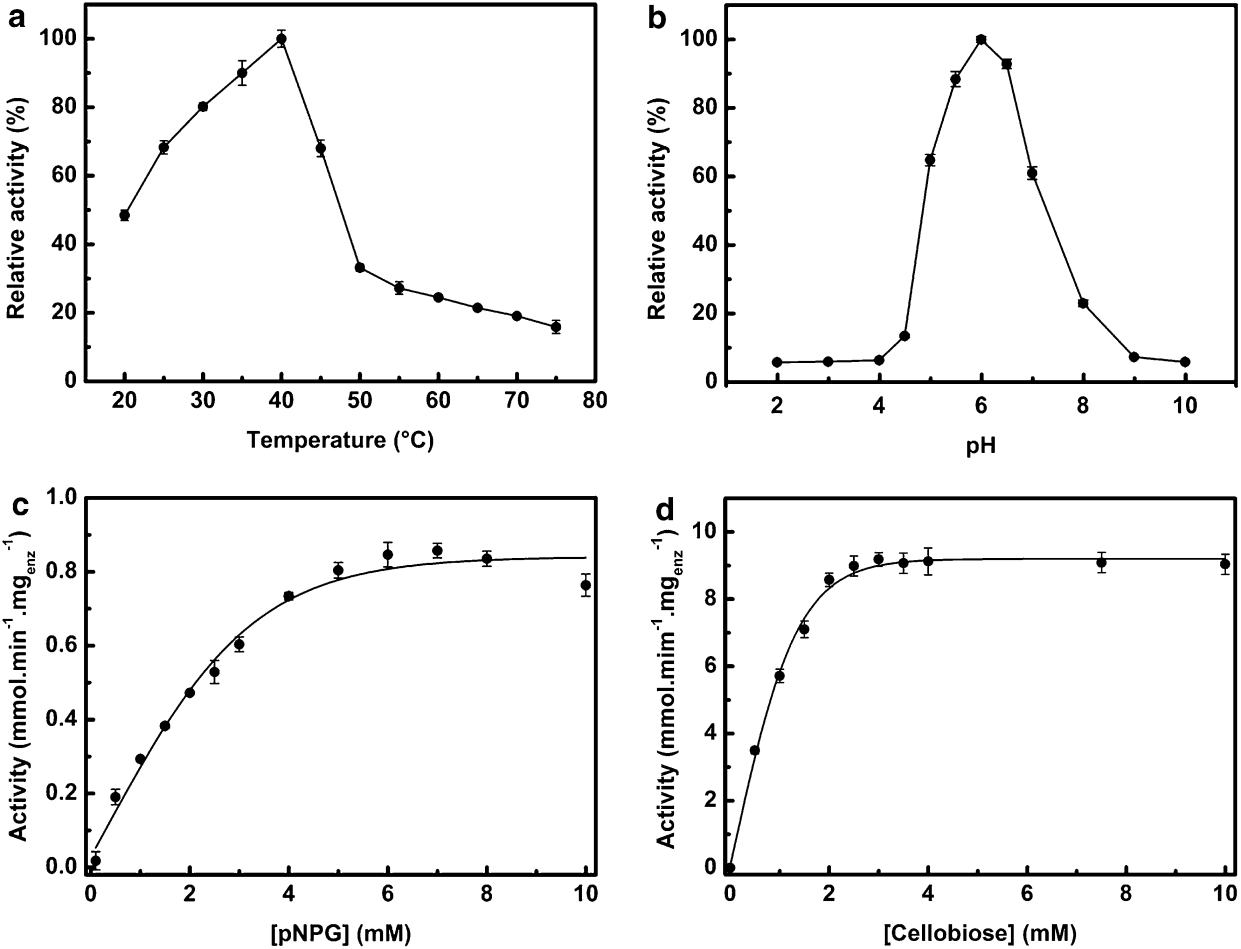
Biochemical properties of purified rThBgl. **a** Optimal temperature screening for rThBgl activity. **b** pH dependence of rThBgl activity in a 100 mM citrate/phosphate/glycine buffer with a pH range from 2 to 10. **c** The kinetic curves of *p*NPG (0–10 mM). **d** The kinetic curves of cellobiose (0–10 mM). For kinetic analysis, the reaction mixture contained 100 mM sodium phosphate buffer pH 6.0, and all reactions were incubated for 10 min at 40 °C

The biochemical characterization of the purified rThBgl was performed using the general artificial substrate for β-glucosidase activity, 4-nitrophenyl β-d-glucopyranoside (*p*NPG). For all enzymatic assays, the N-terminal 6 × His-tag was removed using thrombin to prevent any interference with the enzymatic activity. We first investigated the optimal temperature and pH dependence of the enzymatic activity. Maximal rThBgl activity was observed at 40 °C; however, considerable enzymatic activity was observed in assays performed at temperatures above 50 °C (Fig. 3a). The optimum temperature for β-glucosidase activity has been reported as 25–30 °C for cold-adapted enzymes [41, 42] and 90 °C for thermostable enzymes derived from a metagenomic library of the termite gut [43]. The pH-dependent enzyme activity showed that the rThBgl retains its relative highest activity (>60 %) between pH 5.0 and 7.0, with a catalytic optimum at 6.0 (Fig. 3b). Similar findings were reported for other characterized β-glucosidases [44–46]. Knowledge of the physicochemical characteristics of cellulolytic enzymes is an important step for the development of commercial cocktails designed to improve the enzymatic hydrolysis of lignocellulosic compounds [37, 47].

**Fig. 4:**
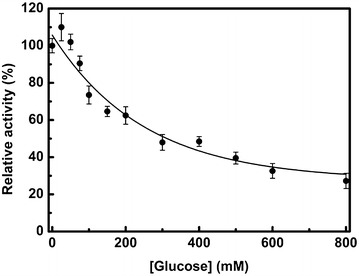
The effect of glucose on rThBgl activity. The glucose tolerance of rThBgl was investigated using the optimal temperature (40 °C) and pH (pH 6.0) for rThBgl activity with glucose concentrations ranging from 0 to 800 mM. The experiments were performed in triplicate

Kinetic parameters obtained with *p*NPG and cellobiose as the substrates under the optimized enzymatic conditions (pH 6.0 and 40 °C) revealed that rThBgl demonstrated typical Michaelis–Menten behavior with a half-saturation constant (*K*_*m*_) and maximum velocity (*V*_*max*_) values of 0.97 mM and 29.3 ± 0.5 U mg_enz_^−1^, respectively, for *p*NPG (Fig. 3c) and 1.22 mM and 10.4 ± 0.6 U mg_enz_^−1^, respectively, for cellobiose (Fig. 3d). A high affinity for *p*NPG is a common characteristic of many β-glucosidases, particularly the aryl-β-glucosidases [3]. The effect of glucose on rThBgl was also investigated (Fig. 4). Although low concentrations of glucose (25–50 mM) had a positive effect and improved the enzymatic activity of rThBgl, a decline in activity was observed at concentrations exceeding 50 mM glucose, and 50 % of the enzymatic activity was observed at 300 mM glucose (Fig. 4). These results indicate that rThBgl is tolerant of product inhibition, but its tolerance is lower compared to other highly glucose-tolerant enzymes, such as HiBG [48]. In sum, its high expression in *T. harzianum* under biomass degradation conditions and its enzymatic properties indicate that rThBgl could be used for supplementation of commercial cocktails with glucose-dependent activity, such as Celluclast, Novozyme N188, and Cellic^®^ CTec2 [41].

### Overall structure of rThBgl

Crystallographic refinement at a resolution of 2.6 Å converged to an *R*_factor_ and *R*_free_ of 0.18 and 0.21, respectively, and resulted in excellent stereochemistry according to Ramachandran and RMSD analyses (Additional file 4: Table S4). rThBgl crystals belonged to the enantiomorphic space group P6_1_ with a dimer in the asymmetric unit and a high solvent content (77.5 %). The two molecules in the asymmetric unit were very similar, with an RMSD for the Cα atoms of 0.15 Å, and each chain comprising the residues Met^1^ to Ala^463^.

**Fig. 5:**
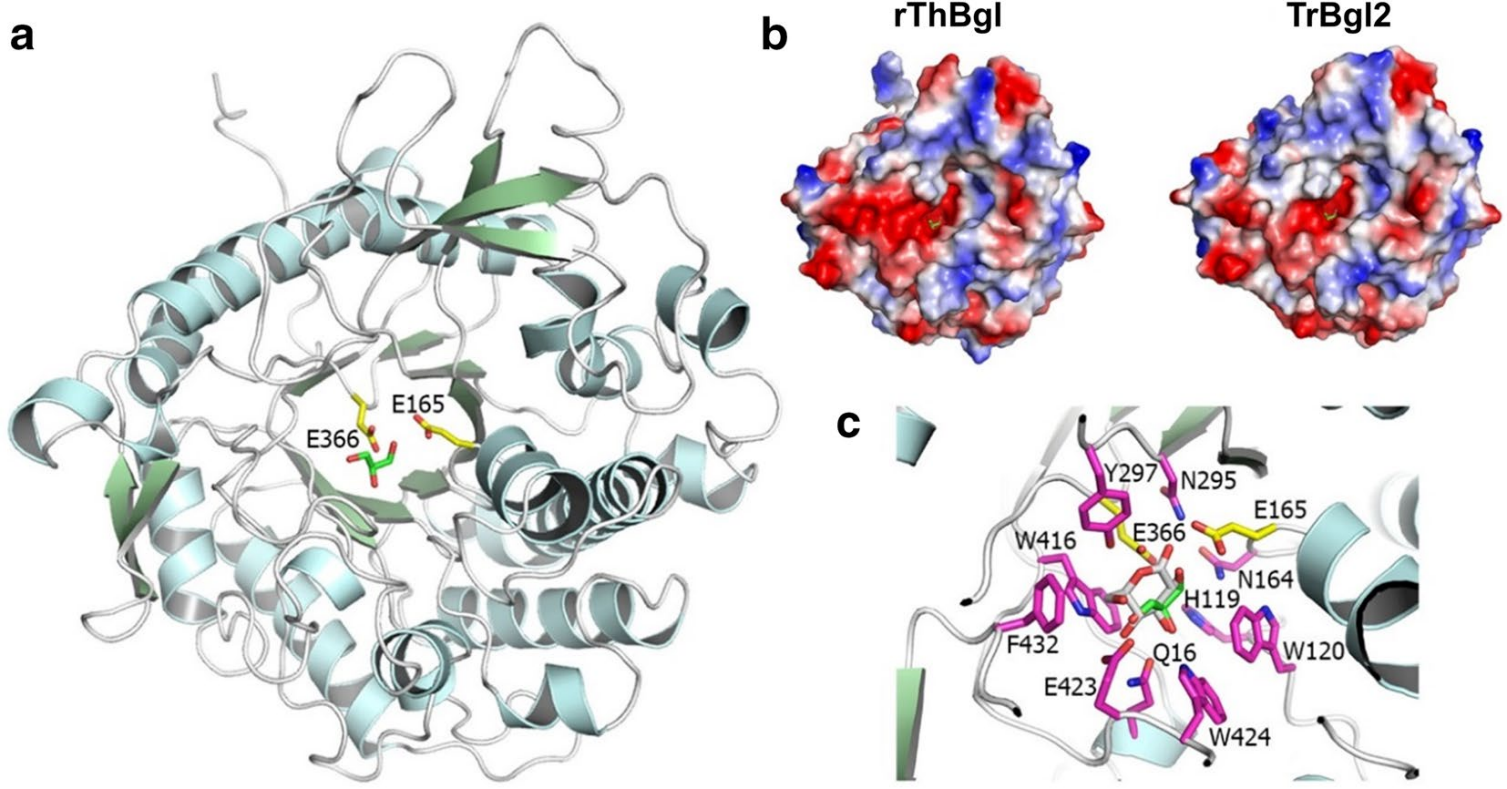
rThBgl structure. **a** Overall molecular architecture highlighting the two acidic catalytic residues and a glycerol molecule at the active-site pocket. **b** Surface charge distribution of the catalytic interface of rThBgl compared to the orthologous TrBgl2 from *Trichoderma reesei*. **c** Conserved residues involved in substrate binding (carbon atoms in *pink*). The gluconolactone molecule from PDB 2E40 (carbon atoms in *white*) and the glycerol molecule (carbon atoms in *green*) are represented as sticks

rThBgl has the classical (α/β)_8_-barrel fold observed in other structurally characterized GH1 β-glucosidases, with the active-site pocket located at the C-terminal region of the barrel (Fig. 5a). rThBgl shares a 90 % sequence identity with TrBgl2 (PDB code 3AHY), and their structural alignment resulted in an RMSD of 0.26 Å with the main differences in the flexible regions, including the *N*- and *C*-termini. The active-site architecture was fully conserved, and the high structural similarity between rThBgl and TrBgl2 [49] was reflected in the surface charge distribution, with a canonical negatively charged active-site pocket (Fig. 5b). By comparison, the Glu^366^ residue is the nucleophile, and the Glu^165^ residue is the acid–base [50]. Based on the superposition with a β-glucosidase from *Phanerochaete chrysosporium* (BGL1A) in a complex with gluconolactone (PDB code 2E40; [51]), other residues involved in substrate binding were also conserved, including Gln^16^, His^119^, Trp^120^, Asn^164^, Asn^295^, Tyr^297^, Trp^416^, Glu^423^, Trp^424^, and Phe^432^ (Fig. 5c). The rThBgl structure also contained a glycerol molecule that mimicked a carbohydrate moiety bound to the active site and interacted with several of the residues considered essential for substrate recognition (Fig. 5c).

**Fig. 6:**
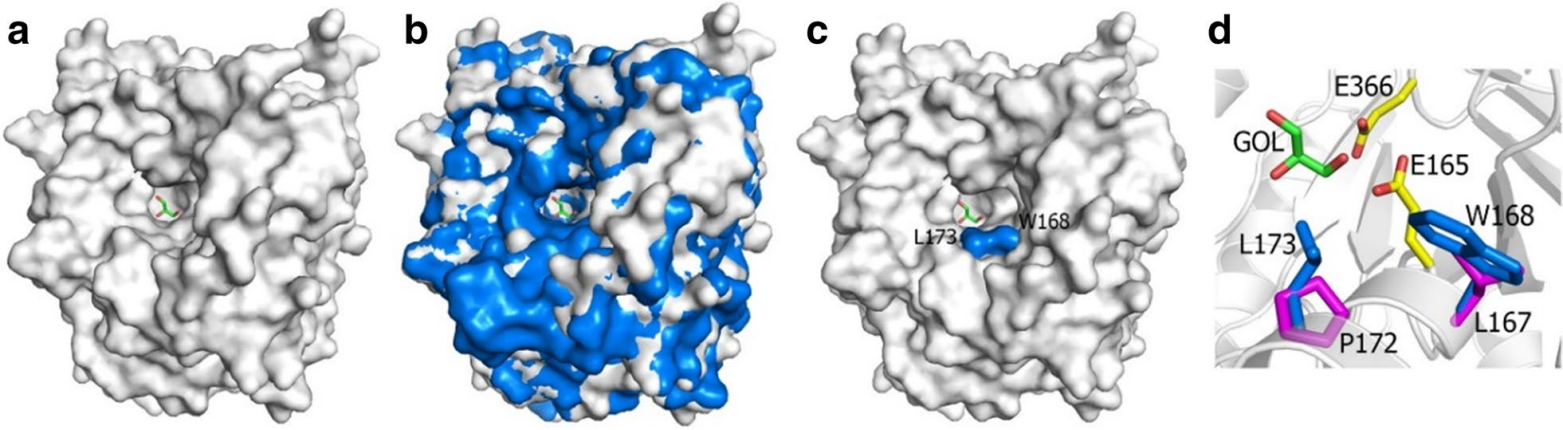
Analysis of active-site accessibility with the glycerol molecule depicted to indicate the active-site pocket. **a** The surface representation of rThBgl. **b** The HiBG surface superimposed on rThBgl. **c** The representation of the two gatekeeper residues (Trp^168^ and Leu^173^) on the rThBgl surface. **d** A stick representation of the two gatekeepers substituted with a Leu^167^ and Pro^172^ in rThBgl

In comparison with the structure of HiBG, a highly glucose-tolerant GH1 β-glucosidase from *Humicola insolens*, rThBgl has a broader active-site entrance, which may explain the lower tolerance of this enzyme to glucose inhibition compared to HiBG (Fig. 6a–c). According to de Giuseppe et al. [48], restricted access to the active-site pocket is associated with the high glucose tolerance of some GH1 β-glucosidases, such as HiBG [48]. Moreover, the two HiBG residues Trp^168^ and Leu^173^ are considered to be gatekeepers involved in glucose tolerance. These two residues were not conserved in rThBgl and were replaced by Leu^167^ and Pro^172^, respectively (Fig. 6c–d).

## Conclusions

In the present study, we used RNA-Seq data mining to identify the β-glucosidases highly expressed by *T. harzianum* under biomass degradation conditions. We used a rational approach to investigate the biochemical characteristics of the β-glucosidase and to obtain a detailed three-dimensional structure. Although high mRNA expression is not necessarily coupled to a high level of the translated protein, up-regulated genes involved in cellulose metabolism suggest that these gene products effectively participate in metabolic pathways. Thus, these enzymes are excellent targets for further investigation into novel biotechnological applications. The strategy described in this study provides a model for the bio-prospection of key enzymes for the development or improvement of enzymatic cocktails designed for ethanol production from plant biomass.

## Methods

### Data mining using RNA-Seq libraries

The RNA-Seq libraries from *T. harzianum* IOC-3844 cultured in the presence of LAC, CEL, or DSB [18] were used to identify the major β-glucosidase used by *T. harzianum* under biomass degradation conditions. These data can be found in the NCBI’s Sequence Read Archive (SRA) under accession numbers SRR579379, SRR631745, and SRR631746 for the DSB, CEL, and LAC libraries, respectively.

The data mining was conducted using the CLC Genomics Workbench (v4.0; Finlandsgade, Dk). The genes encoding the GH1 and GH3 β-glucosidases (Additional file 1: Table S1) used for performing the read mapping were identified in the assembled genome of the *T. harzianum* T6776 strain (GenBank access number JOKZ00000000.1; [24]). For read mapping, the parameters were set to a similarity  =  0.8, length fraction  =  0.9, and maximum number of hits for a read = 10.

To compare the relative gene expression between the different β-glucosidase sequences under three different conditions (LAC, CEL, and DSB), we log_2_-transformed the normalized reads per million mapped values. The squalene epoxidase (GenBank: AM050097.1), TATA box-binding protein (GenBank code: KKP02269.1), and 13-kDa ribonucleoprotein (RNP)-associated protein (GenBank: KKO98059.1) sequences were used for the normalization of gene expression.

### DNA manipulation

The DNA amplification and recombinant plasmid construction were carried out using standard molecular biology procedures [52].

The *bgl* gene (1398 bp) encoding the GH1 β-glucosidase protein was amplified from *T. harzianum* IOC-3844 cDNA using PCR. Following RNA extraction, the cDNA was synthesized using a SuperScript II kit (Invitrogen, USA) according to the manufacturer’s instructions. The primers 5′-TATCATATGTTGCCCAAGGACTTT-3′ and 5′-TATGAATTCTTACTCCTTCGCAATC-3′ contained *Nde*I and *EcoR*I restriction sites (underlined), respectively, and were designed using the sequence information available in GenBank (access number KU201604.1). The PCR amplification product was cloned into a pET28a(+) (Novagen, Madison, WI, USA) expression vector, which added an N-terminal six-histidine tag and a thrombin protease site to the coding sequence. Nucleotide substitutions in the constructed plasmid were analyzed with DNA sequencing.

### Heterologous protein expression and purification

rThBgl was expressed in the *E. coli* Rosetta strain (Novagen, Darmstadt, Germany). The cells were cultured at 37 °C with shaking at 300 rpm in 1 L of LB broth containing chloramphenicol (34 µg mL^−1^) and kanamycin (30 µg mL^−1^) until an OD_600_ of 0.8 was reached. Recombinant protein expression was induced by 0.4 mM IPTG, followed by cultivation for 20 h at 16 °C and 180 rpm. The culture was then centrifuged (3000*g*, 15 min, 4 °C), and the cells were resuspended in 25 mL of buffer A (40 mM HEPES pH 7.5 and 150 mM NaCl) containing 1 mg mL^−1^ lysozyme, 1 mM PMSF (phenylmethanesulfonyl fluoride), and 0.1 % (v/v) Tween 20. The cells were disrupted by sonication, and the soluble fraction was collected by centrifugation (20,000*g*, 40 min, 4 °C). The purification of rThBgl was completed using nickel affinity chromatography with a prepacked Ni Sepharose High Performance HisTrap column (GE Life Sciences) previously equilibrated with buffer A. A polishing purification step was performed using gel filtration with a HiPrep 16/60 Sephacryl S-100 HR column (GE Life Sciences) previously equilibrated with buffer A. All chromatographic steps were carried out with columns coupled to an ÄKTA FPLC device (GE Life Sciences), and the protein elution profiles were monitored at an absorbance of 280 nm. The His_6_-tag of rThBgl was cleaved by treating 10 mg of the target protein with 1 U of thrombin (Novagen, Darmstadt, Germany) for 4 h at 25 °C. The concentrations of the purified proteins were determined spectroscopically using the molar extinction coefficient (*ε*) predicted by the amino acid sequence. The purity of the rThBgl protein was estimated with SDS-PAGE.

### Hydrodynamic and spectroscopic studies

Analytical SEC was performed using a Superdex 200 10/300 GL prepacked column (GE Healthcare, Pittsburgh, PA, USA). Approximately 9 µM protein in buffer A was loaded onto the column at a flow rate of 0.5 mL min^−1^, and the elution profile was monitored at an absorbance of 280 nm. The *R*_s_ of the purified rThBgl protein was estimated using a mix of protein standards with known *R*_s_ values, including carbonic anhydrase (MW = 29 kDa, 23.9 Å), ovalbumin (MW = 44 kDa, 30.5 Å), conalbumin (MW = 75 kDa, 36.4 Å), aldolase (MW = 158 kDa, 48.1 Å), and ferritin (MW = 440 kDa, 61 Å). All of the protein standards (GE Healthcare) were prepared and analyzed under the same conditions used for rThBgl. The Blue Dextran 2000 (GE Healthcare) polymer was used to determine the void volume of the column. The analytical SEC data were calculated according to the manufacturer’s instructions. The estimated *R*_s_ values obtained from the analytical SEC data were used to estimate the *ƒ*/*ƒ*_0_ as the ratio of the experimental *R*_s_ to the predicted radius of a sphere (*R*_0_) of the same molecular mass.

Sedimentation velocity experiments with the rThBgl protein were performed using a Beckman Optima XL-A analytical ultracentrifuge. The data acquisition during the AUC was performed at 280 nm, 20 °C, and 35,000 rpm using an AN-50Ti rotor with a protein sample ranging from 100 to 800 µg mL^−1^ in buffer A. The buffer viscosity (*η* = 1.0513 × 10^−2^ poise), buffer density (*ρ* = 1.0163 g mL^−1^), and partial-specific rThBgl volume (Vbar: rThBgl = 0.733194 mL g^−1^) were estimated using the Sednterp server (http://sednterp.unh.edu/). The *R*_s_, MM_exp_, $S_{20,w}^{0}$*S,* and *ƒ*/*ƒ*_0_ were obtained from the AUC data using SedFit software (Version 12.1).

The rThBgl secondary structure was analyzed via CD with a Jasco model J-815 CD spectropolarimeter (Japan Spectroscopic; Tokyo, Japan) coupled to a thermoelectric sample temperature controller (Peltier Type Control System PFD 425S-Jasco) to record the CD spectra. The far-UV CD spectra were generated using the rThBgl protein at a concentration of approximately 4 µM in buffer A at 25 °C. The assays were performed using a quartz cuvette with a path length of 1 mm. A total of 18 determinations within the range of 260–200 nm at a rate of 20 nm min^−1^ were recorded and averaged. The statistical analysis of the CD spectra was performed using CDNN deconvolution software. The rThBgl thermal-induced unfolding experiments followed by CD were measured at 220 nm from 20 to 90 °C with a 1-mm-path length cell using approximately 8 µM rThBgl in buffer A.

For the fluorescence spectroscopy analyses, a Varian Cary Eclipse fluorescence spectrophotometer (Agilent Technologies; Santa Clara, USA) was used. rThBgl samples (2.5 μM) containing 0 or 6 M GdnHCl were prepared in buffer A. Using a 10 × 2-mm-path-length cell, the samples were excited at 280 nm, and the intrinsic fluorescence emission spectra were collected from 300 to 500 nm. The λ_max_ and 〈*λ*〉 were calculated from the intrinsic fluorescence emission data.

### Biochemical characterization

The β-glucosidase activity of the purified rThBgl was initially evaluated using *p*NPG (Sigma-Aldrich) as the substrate. The initial experiments were performed in triplicate with 100 μL reactions containing 25 nM purified enzyme, 100 mM sodium phosphate buffer, pH 7.0, and 0.5 mM *p*NPG. All reactions were incubated for 10 min and stopped with the addition of 100 μL of 1 M Na_2_CO_3_. The *p*-nitrophenol released during the reaction was measured at 405 nm using an Infinite^®^ 200 PRO microplate reader (TECAN). One unit of enzyme activity was defined as 1 μM of *p*-nitrophenol released per minute.

The optimal temperature was evaluated in assays ranging from 20 to 75 °C. The pH dependence of the enzymatic activity was determined in a pH range from 2.0 to 10.0 using the following buffers: citrate–phosphate (pH 2, 3, 4, 4.5, 5, and 5.5), phosphate (pH 6, 6.5, 7, and 8), and glycine (pH 9 and 10) at a final concentration of 100 mM.

Subsequent to the determination of the optimal temperature and pH, kinetic experiments were performed in 100 mM sodium phosphate buffer (pH 6.0) at 40 °C using the rate of hydrolysis of *p*NPG and cellobiose at various concentrations ranging from 0 to 10 mM. The kinetic parameters (*K*_m_ and *V*_max_) were obtained using GraphPad Prism (GraphPad Software, San Diego, CA, USA) to adjust for the non-linear fit of the Michaelis–Menten equation.

Activity inhibition by glucose was investigated with glucose concentrations ranging from 0 to 800 mM.

### Crystallization, data collection, structure determination, refinement, and validation

The protein was concentrated to 10 mg mL^−1^ in 50 mM phosphate buffer (pH 7.0) for the crystallization experiments. Sitting drops were prepared at 18 °C using a Cartesian HoneyBee 963 system (Genomic Solutions), and 544 conditions were screened based on the commercially available crystallization kits from Hamptom Research (SaltRx, Crystal Screen I and II), Emerald BioSystems (Precipitant Synergy and Wizard I and II), and Qiagen/Nextal (PACT and JCSG+). Suitable crystals for X-ray diffraction experiments were obtained with 2.0 M ammonium sulfate, 0.1 M sodium acetate, pH 5.5, and 2 % (v/v) PEG400. The diffraction data were acquired using the W01B-MX2 beamline (LNLS, Campinas, Brazil). A single crystal was soaked in the aforementioned crystallization conditions with 30 % (v/v) glycerol as a cryoprotectant and then directly flash-cooled in a nitrogen gas stream at 100 K. The sample-to-detector distance was set to a maximum resolution of 2.5 Å, and 180° were collected using the fine-slicing method (0.1° per image) and a Pilatus 2 M detector (Dectris). The data were indexed, integrated, and scaled using the XDS package [53, 54]. Molecular replacement calculations were performed using the program MOLREP [55], and the structure of β-glucosidase 2 from the fungus *T. reesei* (TrBgl2, PDB code 3AHY; [49]) was used as a template. Restrained refinement was completed with the phenix.refine program from the PHENIX package [56], and manual inspection and building was performed with COOT [57]. The model quality was assessed using MOLPROBITY [58], and the refinement statistics are presented in Additional file 4: Table S4. The atomic coordinates and structure factors have been added to the PDB under the accession code 5BWF.

## Additional files

10.1186/s13068-016-0487-0 GH1 and GH3 β-glucosidases sequences identified on *T. harzianum* T6776 genome (GenBank access number JOKZ00000000.1) and used for mapping experiments via RNA-Seq data.
10.1186/s13068-016-0487-0 Percent identity matrix between the GH1 β-glucosidases amino acid sequences. The multiple sequence alignment was performed using the Clustal Omega server (http://www.ebi.ac.uk/Tools/msa/clustalo/).
10.1186/s13068-016-0487-0 Percent identity matrix between the GH3 β-glucosidase. The multiple sequence alignment was performed using the Clustal Omega server (http://www.ebi.ac.uk/Tools/msa/clustalo/).
10.1186/s13068-016-0487-0 Data collection and refinement statistics.

## Abbreviations

rThBgl: recombinant β-glucosidase of *T. harzianum*
GH: glycosyl hydrolase
PDB: protein data bank
RNA-Seq: RNA sequencing
RPKM: reads per kilo base per million of mapped reads
LAC: lactose
CEL: crystalline cellulose
DSB: delignified sugarcane bagasse
SEC: size-exclusion chromatography
AUC: analytical ultracentrifugation
MM_prep_: predicted molecular mass
MM_app_: apparent molecular mass
MM_exp_: experimental molecular mass
*R*_0_: predicted radius of a sphere
*R*_s_: stokes radius
*ƒ*/*ƒ*_0_: frictional ratio
CD: circular dichroism
*λ*_max_: maximum emission wavelength
〈λ〉: spectral center of mass
*p*NPG: 4-nitrophenyl β-d-glucopyranoside
*K*_m_: half-saturation constant
*V*_max_: maximum velocity
