# Supplementary material for: Crystal structure and biochemical characterization of the recombinant ThBgl, a GH1 β-glucosidase overexpressed in Trichoderma harzianum under biomass degradation conditions
Source: Biotechnol Biofuels. 2016 Mar 22;9:71. doi: 10.1186/s13068-016-0487-0 (PMC4802607; doi:10.1186/s13068-016-0487-0)
Supplement: Supplementary file 1 — 10.1186/s13068-016-0487-0 GH1 and GH3 β-glucosidases sequences identified on T. harzianum T6776 genome (GenBank access number JOKZ00000000.1) and used for mapping experiments via RNA-Seq data. [file 13068_2016_487_MOESM1_ESM.pdf]

Table S1. GH1 and GH3 β-glucosidases sequences identified on *T. harzianum* T6776 genome (GenBank access number **JOKZ00000000.1**) and used for mapping experiments via RNA-Seq data.

GenBank acession number	Locus tag	Product name	Product length
Glycosyl hydrolase family 1			
**KKP02477.1**	THAR02_05432	beta-glucosidase	465 aa
**KKP05610.1**	THAR02_02251	beta-glucosidase	484 aa
**KKO98105.1**	THAR02_09788	beta-glucosidase	622 aa
**KKP06709.1**	THAR02_01198	beta-glucosidase A	642 aa
Glycosyl hydrolase family 3			
**KKO97043.1**	THAR02_10851	beta-glucosidase	781 aa
**KKO97705.1**	THAR02_10190	beta-glucosidase	835 aa
**KKP07226.1**	THAR02_00656	beta-glucosidase	770 aa
**KKP00605.1**	THAR02_07292	beta-glucosidase	882 aa
**KKP05725.1**	THAR02_02189	beta-glucosidase	767 aa
**KKP04308.1**	THAR02_03585	beta-glucosidase K	839 aa
