# Supplementary material for: Crystal structure and biochemical characterization of the recombinant ThBgl, a GH1 β-glucosidase overexpressed in Trichoderma harzianum under biomass degradation conditions
Source: Biotechnol Biofuels. 2016 Mar 22;9:71. doi: 10.1186/s13068-016-0487-0 (PMC4802607; doi:10.1186/s13068-016-0487-0)
Supplement: Supplementary file 2 — 10.1186/s13068-016-0487-0 Percent identity matrix between the GH1 β-glucosidases amino acid sequences. The multiple sequence alignment was performed using the Clustal Omega server (http://www.ebi.ac.uk/Tools/msa/clustalo/). [file 13068_2016_487_MOESM2_ESM.pdf]

Table S2. Percent identity matrix between the GH1 β-glucosidases amino acid sequences. The multiple sequence alignment was performed using the Clustal Omega server (http://www.ebi.ac.uk/Tools/msa/clustalo/).

	**KU201604**	**KKP02477.1**	**KKP05610.**	**KKO98105.1**	**KKP06709.1**
**KU201604**	**100.00**	99.57	53.78	31.04	29.02
**KKP02477.1**	99.57	**100.00**	53.78	31.28	29.02
**KKP05610.1**	53.78	53.78	**100.00**	30.86	27.46
**KKO98105.1**	31.04	31.28	30.86	**100.00**	50.16
**KKP06709.1**	29.02	29.02	27.46	50.16	**100.00**
