# Supplementary material for: Crystal structure and biochemical characterization of the recombinant ThBgl, a GH1 β-glucosidase overexpressed in Trichoderma harzianum under biomass degradation conditions
Source: Biotechnol Biofuels. 2016 Mar 22;9:71. doi: 10.1186/s13068-016-0487-0 (PMC4802607; doi:10.1186/s13068-016-0487-0)
Supplement: Supplementary file 3 — 10.1186/s13068-016-0487-0 Percent identity matrix between the GH3 β-glucosidase. The multiple sequence alignment was performed using the Clustal Omega server (http://www.ebi.ac.uk/Tools/msa/clustalo/). [file 13068_2016_487_MOESM3_ESM.pdf]

Table S3. Percent identity matrix between the GH3 β-glucosidase. The multiple sequence alignment was performed using the Clustal Omega server (http://www.ebi.ac.uk/Tools/msa/clustalo/).

	**KKP07226.1**	**KKO97705.1**	**KKP04308.1**	**KKP00605.1**	**KKO97043.1**	**KKP05725.1**
**KKP07226.1**	**100.00**	30.05	28.88	25.15	23.96	25.30
**KKO97705.1**	30.05	**100.00**	46.83	30.35	31.63	31.18
**KKP04308.1**	28.88	46.83	**100.00**	26.44	28.61	27.58
**KKP00605.1**	25.15	30.35	26.44	**100.00**	41.58	40.54
**KKO97043.1**	23.96	31.63	28.61	41.58	**100.00**	50.00
**KKP05725.1**	25.30	31.18	27.58	40.54	50.00	**100.00**
