# Supplementary material for: Crystal structure and biochemical characterization of the recombinant ThBgl, a GH1 β-glucosidase overexpressed in Trichoderma harzianum under biomass degradation conditions
Source: Biotechnol Biofuels. 2016 Mar 22;9:71. doi: 10.1186/s13068-016-0487-0 (PMC4802607; doi:10.1186/s13068-016-0487-0)
Supplement: Supplementary file 4 — 10.1186/s13068-016-0487-0 Data collection and refinement statistics. [file 13068_2016_487_MOESM4_ESM.pdf]

Table S4. Data collection and refinement statistics.

Wavelength (Å)	1.458
Resolution range (Å)	47.25 – 2.59 (2.69 – 2.59)
Space group	$P\ 6_1$
Unit cell parameters (Å)	a = b = 218.24, c = 84.67
Total reflections	599,938 (42,039)[*]
Unique reflections	70,851 (6,926)
Multiplicity	8.5 (6.1)
Completeness (%)	99.79 (98.54)
Mean I/sigma (I)	9.83 (2.00)
Wilson B-factor	30.63
R-merge	0.234 (0.908)
R-meas	0.249
$CC_{1/2}$	0.98 (0.68)
CC*	0.99 (0.89)
R-work	0.176 (0.268)
R-free	0.210 (0.317)
Number of non-hydrogen atoms	7,803
Macromolecules	7,604 (2 chains)
Ligands	38 (3 GOL / 4 SO4)
Water	161
Protein residues	942
RMS (bonds) (Å)	0.003
RMS (angles) (°)	0.70
Ramachandran favoured (%)	96
Ramachandran outliers (%)	0
Clashscore	3.69
Average B-factor	37.30
Macromolecules	37.30
Ligands	62.80
Solvent	31.50
PDB accession code	5BWF

[*]Statistics for the highest-resolution shell are shown in parentheses.
